# Impact of thermal stress on evolutionary trajectories of pathogen resistance in three-spined stickleback (*Gasterosteus aculeatus*)

**DOI:** 10.1186/s12862-014-0164-5

**Published:** 2014-07-26

**Authors:** Franziska M Schade, Lisa NS Shama, K Mathias Wegner

**Affiliations:** 1 Alfred Wegener Institute Helmholtz Centre for Polar and Marine Research, Hafenstrasse 43, List/Sylt, 25992, Germany

**Keywords:** Climate change, Infectious diseases, Vibrio tubiashii, Genotype x environment Interaction, Selection

## Abstract

**Background:**

Pathogens are a major regulatory force for host populations, especially under stressful conditions. Elevated temperatures may enhance the development of pathogens, increase the number of transmission stages, and can negatively influence host susceptibility depending on host thermal tolerance. As a net result, this can lead to a higher prevalence of epidemics during summer months. These conditions also apply to marine ecosystems, where possible ecological impacts and the population-specific potential for evolutionary responses to changing environments and increasing disease prevalence are, however, less known. Therefore, we investigated the influence of thermal stress on the evolutionary trajectories of disease resistance in three marine populations of three-spined sticklebacks *Gasterosteus aculeatus* by combining the effects of elevated temperature and infection with a bacterial strain of *Vibrio* sp*.* using a common garden experiment.

**Results:**

We found that thermal stress had an impact on fish weight and especially on survival after infection after only short periods of thermal acclimation. Environmental stress reduced genetic differentiation (Q_ST_) between populations by releasing cryptic within-population variation. While life history traits displayed positive genetic correlations across environments with relatively weak genotype by environment interactions (GxE), environmental stress led to negative genetic correlations across environments in pathogen resistance. This reversal of genetic effects governing resistance is probably attributable to changing environment-dependent virulence mechanisms of the pathogen interacting differently with host genotypes, i.e. G_Pathogen_xG_Host_xE or (G_Pathogen_xE)x(G_Host_xE) interactions, rather than to pure host genetic effects, i.e. G_Host_xE interactions.

**Conclusion:**

To cope with climatic changes and the associated increase in pathogen virulence, host species require wide thermal tolerances and pathogen-resistant genotypes. The higher resistance we found for some families at elevated temperatures showed that there is evolutionary potential for resistance to *Vibrio* sp. in both thermal environments. The negative genetic correlation of pathogen resistance between thermal environments, on the other hand, indicates that adaptation to current conditions can be a weak predictor for performance in changing environments. The observed feedback on selective gradients exerted on life history traits may exacerbate this effect, as it can also modify the response to selection for other vital components of fitness.

## Background

Pathogens are strong selective agents that can influence biodiversity by regulating host population dynamics [[1],[2]]. Environmental conditions can amplify these effects [[3]], and many infectious diseases are temperature-dependent [[4]]. An increase in ambient temperature will affect the pathogen by, for example, enhancing its metabolism resulting in faster development and a higher number of transmission stages per generation. Especially for many bacterial disease agents, population growth rates increase exponentially with increasing temperature [[5],[6]]. Since population growth rate closely resembles fitness for prokaryotes, improved pathogen fitness can also lead to a higher prevalence of diseases [[7]].

Rising temperatures are also likely to stress the host organism, thereby increasing host susceptibility to diseases [[8],[9]]. This also applies to marine organisms [[10]], which are often highly vulnerable to climatic changes [[11]]. Thermal stress causes an alteration of the immune response, which generally results in a reduction of pathogen resistance [[12]]. Despite these multifaceted effects, the specific interactions of increased thermal stress due to climatic warming with infectious diseases are sparsely explored, particularly in marine ecosystems.

The ecological and evolutionary consequences of thermal stress-disease interactions will strongly depend on the thermal tolerance and rate of adaptation of populations to climate warming [[13]]. A high potential for acclimation within and between generations and the capacity for adaptation by selection of pathogen-resistant genotypes will ensure population viability and biodiversity [[14],[15]]. Species with rapid turnover of generations are likely to adapt fast enough to cope with the changing environment [[16]]. For vertebrates, the potential for adaptation by microevolution is considered to be alarmingly low [[17]]. Standing genetic variation supplies the raw material for adaptation, and different genotypes will show different reaction norms leading to genotype by environment (GxE) interactions. GxE interactions as well as phenotypic plasticity can potentially compensate for declines in mean fitness of populations with low evolutionary potential. Furthermore, it also has been shown that changing environmental conditions can release otherwise cryptic genetic variation [[18]], which can consequently alter the evolutionary potential of a population.

Here, we tested whether environmental change can also alter the genetic components of resistance to pathogen challenges, and whether such changes can feed back on selection gradients of other components of fitness. To do so, we set up a common garden breeding experiment using three populations of marine three-spined sticklebacks *Gasterosteus aculeatus* and challenged the offspring with an infection by a bacterial strain of *Vibrio* sp*.* in two different temperature environments (ambient and stressful). In general, bacteria of the genus *Vibrio* include many facultative symbionts and pathogenic strains [[19]]. They are strongly temperature-dependent with elevated population sizes during summer [[20],[21]]. Moreover, they are able to adapt rapidly to environmental changes due to high genome plasticity including frequent mutation, recombination and lateral gene transfers [[22]], making them ideal candidates for our purpose to study host-parasite interactions in changing environments. *Vibrio tubiashii*, in particular, has been described as a pathogen of bivalves [[23],[24]], but is also known to harm several marine fish species [[25],[26]], where Vibriosis can lead to superficial skin lesions, muscle necrosis and haemorrhages which are likely to cause mortality [[27]].

The three-spined stickleback represents an ideal model system to study the evolutionary ecology of host-parasite interactions [[28]-[30]] especially in connection with environmental change. These fish occur in shallow freshwater and marine habitats with a temperature range from 4 to 20°C [[31]], but show preferences for intermediate temperatures (15-18°C [[32],[33]]). Our experimental temperature environments were set to simulate the average summer temperature of coastal North Sea areas (i.e. 17°C) and elevated temperatures with reference to recent climate change predictions for the North Sea (21°C) [[34]], which was shown to result in diminished growth rates after long-term exposure in one of the populations used here [[35]].

With this study, we estimated the effects of thermal stress on pathogen resistance (i.e. survival after infection) and its interactions with other life history traits that can affect an individual’s survival and reproductive potential. In a narrower sense, life history traits include gestation time, age to sexual maturity, reproductive life span and number of progeny. In a wider sense, morphological traits such as length and weight can also be seen as life history traits representing key maturational and reproductive characteristics, and we thus chose to also measure size and weight.

Short-term fluctuations and extreme events like heat waves that are predicted to increase in the nearer future [[36]] could have very different effects on populations with different adaptive thermal optima. By additionally determining the within- and between-population genetic components of resistance and life history traits, we can also project if environmental stress will modify the results of population-specific evolutionary trajectories by releasing or absorbing genetic variance for traits under selection. The connection between environmentally modified pathogen-induced selection to its consequences for other components of fitness will help to deepen our understanding of how populations can or cannot cope with increasingly stressful and pathogen rich environments.

## Methods

### Sampling and fish breeding

During April and June 2011, adult marine sticklebacks were caught by dip netting in an oyster farm in Yerseke (Oosterschelde, The Netherlands, 51.49 N, 4.06 E) and in tidal channels on the islands of Texel (The Netherlands, 53.16 N, 4.88 E) and Sylt (Germany, 55.03 N, 8.46 E). Between 50–70 individuals were sampled from each location and transferred in groups of 5–7 fish to 20 l aquaria (38 × 25 × 20 cm) equipped with permanent seawater flow-through at a constant temperature of 17°C. Between July and August, laboratory-bred F1 families were produced for each population (20 families per population) using a modified North Carolina II breeding design (five replicates of crosses between two dams x two sires i.e. four half-sibling families). Since not all of the crosses resulted in successful hatchings, we produced additional families (n = 10 and using different parents) to increase sample size and achieve better estimates of within-population variation for the Sylt population. Eggs were kept in aerated 1 l glass beakers until hatching. Hatchlings were then transferred to 10 l flow through tanks (19 × 25 × 20,5 cm) with a water temperature of 17°C and a 16 h: 8 h light: dark cycle. In total, 14 families from Oosterschelde, 11 families from Texel and 24 families from Sylt hatched.

One month after hatching, the density of each family was reduced to 20 fish per 10 l aquaria. Fish were fed daily *ad libitum* with live *Artemia* sp. until 12 weeks of age and afterwards with frozen chironomid larvae until the end of the experiment. During the early rearing phase, we lost several families due to electrical failure of aeration pumps. These affected only newly hatched families from all populations and did not lead to a systematic bias in mortality (χ^2^_2_ = 0.584, *P* = 0.747). In the end, nine families remained from the Oosterschelde population (n_fish_ = 164), nine families from Texel (n_fish_ = 192) and 13 families from Sylt (n_fish_ = 263) that had sufficient family sizes (20 fish per family) for the infection experiment.

### Infection experiment

Eight months after hatching, families were separated into smaller groups of 10 fish per 2,6 l aquarium (12 × 18,5 × 11,8 cm). In line with recent climate change predictions for the North Sea [[34]], half of each family (n = 10) was experimentally exposed to elevated temperatures by increasing the water temperature up to 21°C. The remaining half (n = 10) was kept at their rearing temperature of 17°C as a control. After a four week thermal acclimation period, fish from both temperature treatments were injected either with 10 μl of a solution containing a bacterial strain of *Vibrio* sp. closely related to a pathogenic strain of *Vibrio tubiashii* (10^7^ cells per ml, 17°C n = 5, 21°C n = 5) or with the same amount of phosphate buffered saline (PBS) medium as control (17°C n = 5, 21°C n = 5). Infected and control fish were then held in ambient or elevated water temperature without flow through and susceptibility was determined by monitoring induced mortality every 12 hours for 10 days. Dead fish as well as fish remaining at the end of the experiment were weighed (wet mass to the nearest 0.01 g) and measured (standard length to the nearest 0.1 cm). All experiments were conducted in accordance with German animal welfare laws (Permission No. V312-72241.123-16).

### Microsatellite genotyping

To measure drift-based neutral divergence between populations, each parental fish (n = 70) was genotyped at 15 microsatellite loci. DNA was extracted from the caudal fin using the DNAeasy Blood and Tissue Kit (Qiagen, Hilden). The 20 μl multiplex PCR reactions consisted of 4 μl of 5x PCR Flexi buffer, 2 μl 10 mM dNTP, 0.6 μl 25 mM MgCl_2_, 0.1 μl 500 U GoTaq DNA Polymerase (all PCR chemicals from Promega, Mannheim), 5 pmol of each fluorescently labelled forward and unlabelled reverse primer and 2 μl DNA template. Thermal cycling for PCR group 1 (containing markers 5196 HEX, 4170 6_FAM, 1125 6_FAM, 1097 NED and 7033 NED, [[37]]) started with an initial denaturation step at 94°C for 3 min followed by 30 cycles of 94°C for 1 min, 58°C for 1 min and 72°C for 1 min. PCR group 2 (containing markers STN 18 HEX, STN 32 6_FAM, STN 75 HEX and STN 84 NED) started with an initial denaturation step at 94°C for 3 min followed by 30 cycles of 94°C for 45 sec, 56°C for 45 sec and 72°C for 1 min. PCR group 3 (containing markers STN 2 6_FAM, STN 170 HEX and STN 174 HEX) and as well as PCR group 4 (STN 36 6_FAM, STN 114 6_FAM and STN 167 HEX, [[38]]) started with an initial denaturation step at 94°C for 2 min followed by 30 cycles of 94°C for 45 sec, 56°C for 45 sec, 72° for 45 sec and a final extension step at 72°C for 20 min.

One μl of each PCR reaction was denatured in HiDi-formamide (Life Technologies, Darmstadt) at 94°C for 2 min. Fragments were separated using an ABI prism 3100 XL (Life Technologies, Darmstadt) capillary sequencer. Genotype scoring was performed using GeneMarker software (SoftGenetics, version 1.91), and neutral genetic differentiation (measured as F_ST_) was calculated after Weir and Cockerham [[39]] using the software GENETIX [[40]]. Significance of the observed estimates was calculated by bootstrapping using 1000 random permutations of the data. The range for the neutral marker-based differentiation in the Q_ST_-F_ST_ comparison was determined by using the extremes of the distribution of single-locus estimates of F_ST_ for all 15 loci [[41]].

### Statistical analyses

We used generalized linear mixed models (GLMM) using the MCMCglmm package [[42]] of the R statistical environment [[43]] for all of our statistical analyses. First, we wanted to determine the effects of temperature and infection as fixed effects on length, weight and survival using population and full-sibling family as random effects. Density and age were entered as covariates in all models to account for differences among families prior to our experimental treatment. We fitted models as generalized linear mixed models with length and weight as Gaussian and survival as binomial response variables. Markov chains were run for 500’000 iterations and we kept every 100th value after removing 300’000 iterations of burn-in to generate posterior distributions of random and fixed parameters. Model fits were assessed by their respective Deviance Information Criterion (DIC) scores [[44]] including random effects. We used weak but informative priors of half the observed phenotypic variance and examined posterior samples from the Markov chain for signs of autocorrelation. To test for GxE interactions, we additionally fitted random slopes for random terms (singly and combined), and assessed model fit by DIC scores taking a better model fit for a full-sibling family random slopes model as an indication for GxE interactions.

Second, we used population and animal variance components from environment and trait specific GLMMs to calculate population and genetic differentiation in different environments. Divergence in quantitative traits (Q_ST_) was calculated as [[45]],

(1) $$Q_{\mathrm{ST}}=\frac{\sigma_{B}^{2}}{\sigma_{B}^{2}+2\sigma_{W}^{2}}$$

where $\sigma_{B}^{2}$ and $\sigma_{W}^{2}$ are the between- and within-population components of genetic variation. The animal variance component was taken as the measure of within-population variance, while the population variance component was taken as a measure of between-population variation.

We used a character state approach [[46]], treating life history traits and resistance in the different thermal environments as separate traits to calculate genetic correlations of traits across environments. Since individuals could only be assayed in one environment, we did not estimate covariance on the individual/unit level (by using the idh(trait):units covariance structure implemented in MCMCglmm). Variance and covariance for calculating genetic correlations (rG) were estimated on the level of the pedigree by using the us (trait):animal variance components. Genetic correlations across environments were calculated as the covariance between traits divided by the square root of the product of both trait variances. Significance of genetic correlations across environments was assessed by estimating the proportion of estimates from the posterior distributions that overlapped with zero.

The effect of selection gradients imposed on life history traits by bacterial infection was analysed using only data from infected fish in separate thermal environments. GLMMs were fitted with weight or length as response variables, survival status as a fixed effect, density as a covariate, and population and animal as random effects.

## Results

### Effects of thermal stress on life history traits and pathogen resistance

**Figure 1:**
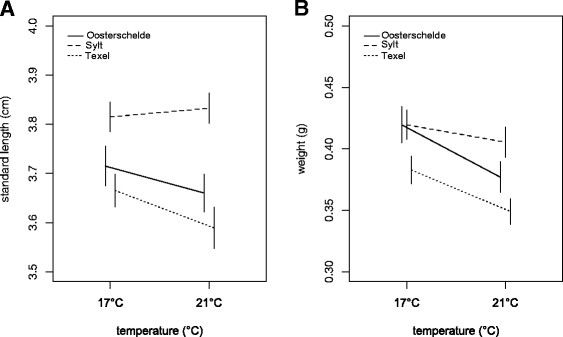
**Effect of thermal stress on life history traits of different stickleback populations.** Lines represent means (with standard errors) of length **(A)** and weight **(B)** of three different populations connecting different temperature treatments. Data from all fish were used.

Thermal stress was visible in life history traits of sticklebacks after the acclimation and experimental period (Figure 1). Fish were on average smaller and lighter at 21°C than at 17°C, but temperature only had significant effects on weight (Table 1). Models fitting separate intercepts but common slopes for populations showed a superior fit, indicating that the effect of temperature on weight was not significantly different between populations, whereas length and weight reached different levels in the populations (random intercepts, Table 1). Although there was considerable variation within families between thermal environments, random slope models did not produce superior model fits, indicating that GxE interactions were comparatively weak. Infection showed no significant effects on life history traits, probably owing to the short time period of the infection experiment (Table 1).

**Table 1:** Summary of the effects of thermal stress on life history traits and pathogen resistance

	**Response**	**Length**		**Weight**		**Survival**	
		**DIC**		**DIC**		**DIC**	
Random	Family (F)	1589.39		1457.55		702.94	
	Population (P) + F	**1586.78**		**1453.26**		539.37	
	P + F (rs)	1604.12		1465.05		**507.26**	
	P (rs) + F (rs)	1606.54		1467.54		507.76	
		Estimate	P	Estimate	P	Estimate	P
Fixed	(Intercept)	**−6.915**	**0.001**	**−8.963**	**<0.001**	0.696	0.573
	Density	**−0.084**	**<0.001**	**−0.118**	**<0.001**	0.007	0.895
	Age	**0.034**	**<0.001**	**0.045**	**<0.001**	−0.042	0.467
	Temperature: 21°C	−0.035	0.628	**−0.163**	**0.004**	**−1.137**	**0.011**
	Infection: Non	−0.058	0.469	−0.037	0.583	**4.156**	**<0.001**

Generalized linear mixed models containing dependent variables length, weight (both Gaussian) and survival (binomial) with temperature and infection as fixed effects and density as a covariate. All models contained population and full-sibling family as random effects. Random slope models (rs) were fitted for both random factors and were retained when they provided better model fit judged by the Deviance Information Criterion (DIC). Fixed effect tables are based on the best fitting model. Best fitting models and significant effects are shown in bold.

**Figure 2:**
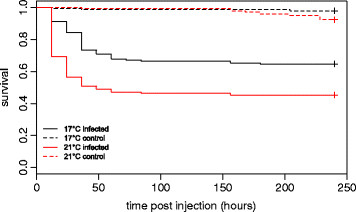
**Survival plot of sticklebacks from different temperature treatments after infection with** ***Vibrio sp.*** **or PBS medium.** Lines show means for all populations pooled.

Infection strongly reduced survival (Figure 2). There was a significant difference between the survival of fish injected with PBS medium and fish injected with the *Vibrio* sp*.* isolate (Table 1). Control fish showed low levels of mortality towards the end of the experiment. Furthermore, temperature had strong effects on survival (Table 1). Infected fish held at the elevated water temperature (21°C) died faster (30.7% mortality within the first 12 hours of the experiment) and in higher numbers (54.6% total mortality after 10 days post injection) than infected fish at 17°C (8.8% mortality in the first 12 hours, 35.2% total mortality after 10 days post injection). Superior fit of models containing random intercepts between populations and random slopes between families indicated that also survival rates differed among populations, and that reaction norms of survival rates varied significantly among families, thus representing GxE interactions (Table 1).

### Effects of thermal stress on quantitative genetic variation

We calculated the drift-based neutral divergence between populations for all populations combined (global F_ST_ = 0.00825, range = −0.00055-0.03901) as well as for each population pair (pairwise F_ST_, Sylt vs. Texel F_ST_ = 0.00339, *P* = 0.19, Sylt vs. Oosterschelde F_ST_ = 0.00926, *P* = 0.01, Oosterschelde vs. Texel F_ST_ = 0.02187, *P* = 0.003). We observed small, but significant neutral differentiation between the Wadden Sea populations (Sylt and Texel) and the Oosterschelde. Our F_ST_ estimates were somewhat smaller than previously reported F_ST_ values for marine stickleback populations (range of 0.08 to 0.19 [[47]-[49]]), but reflected results obtained for other marine fish from the Wadden Sea [[50]].

**Figure 3:**
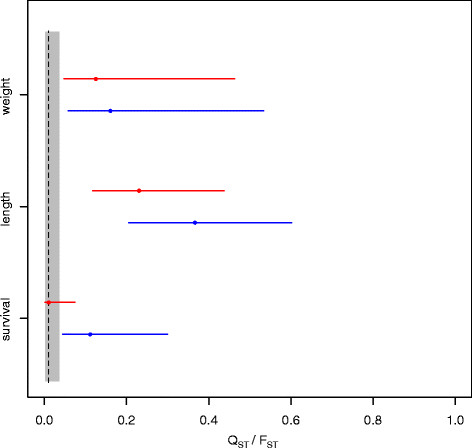
**Quantitative trait and neutral marker differentiation of stickleback populations at different temperature treatments.** The grey box represents the distribution range of global F_ST_ estimates. Blue lines represent 95% confidence intervals of Q_ST_ values for all phenotypic traits at 17°C, while red lines represent 21°C. Dots indicate position of highest densities of posterior distributions. All data were used for weight and length; only data from infected fish were used for survival.

For all investigated traits, quantitative genetic differentiation (Q_ST_) exceeded the overall neutral expectation (F_ST_, Figure 3) at 17°C, suggesting directional selection is driving populations to different phenotypic optima at ambient temperatures [[51]]. Q_ST_ values were on average lower at elevated temperature (21°C, red lines in Figure 3) than at ambient temperature (17°C, blue lines in Figure 3) for all traits, owing to a trend showing a release of within-population genetic variance of the selected traits (V_Animal_, Table 2). This result was most obvious for survival, where Q_ST_ values at elevated temperatures did not exceed neutral expectations, and divergent selection was only visible at ambient temperature. Differences in genetic differentiation between environments were smaller for weight than for length and survival, probably reflecting a stronger environmental influence on this trait that was also visible in the smaller unit variance components, capturing residuals and environmental variation (Figure 2, Table 2).

**Table 2:** Estimated variance components of different traits and temperature treatments

		**17°C**	**21°C**
**Weight**	**17°C**	V_A_ 0.592 (0.262 – 1.028)	Cov 0.862 (0.552 – 1.158)
		V_pop_ 0.216 (0.065 – 1.156)	r_G_ 0.791 (0.641 – 0.891)
		V_U_ 0.375 (0.128 – 0.578)	P _rG=0_ < 0.001
		h^2^ 0.466 (0.137 – 0.713)	
	**21°C**		V_A_ 0.785 (0.478 – 1.036)
			V_pop_ 0.214 (0.077 – 1.185)
			V_U_ 0.167 (0.068 – 0.345)
			h^2^ 0.607 (0.298 – 0.794)
**Length**	**17°C**	V_A_ 0.210 (0.094 – 0.568)	Cov 0.386 (0.295 – 0.810)
		V_pop_ 0.269 (0.081 – 1.585)	r_G_ 0. 821 (0.534 – 0. 905)
		V_U_ 0.698 (0.467 – 0.866)	P _rG=0_ < 0.001
		h^2^ 0.183 (0.055 – 0.402)	
	**21°C**		V_A_ 0.478 (0.264 – 0.946)
			V_pop_ 0.282 (0.097 – 1.699)
			V_U_ 0.453 (0.181 – 0.602)
			h^2^ 0.345 (0.143 – 0.647)
**Survival**	**17°C**	V_A_ 0.834 (0.239 – 7.563)	Cov −2.655 (−377.98 – -0.387)
		V_pop_ 0.351 (0.055 – 5.407)	r_G_ –0.884 (−0.992 – -0.073)
		V_U_ 1*	P _rG=0_ < 0.001
		h^2^ ne*	
	**21°C**		V_A_ 6.604 (0.616 – 100.668)
			V_pop_ 0.701 (0.203 – 4.261)
			V_U_ 1*
			h^2^ ne*

Variance components for V_Animal_, V_Population_, V_Unit_ (representing residual variation including environmental effects) and resulting heritability (h^2^) as well as genetic correlations across environments (r_G_) with 95% confidence intervals. Estimates are based on standardized values. *fixed residual variation because categorical traits were used and heritabilities were not estimable.

**Figure 4:**
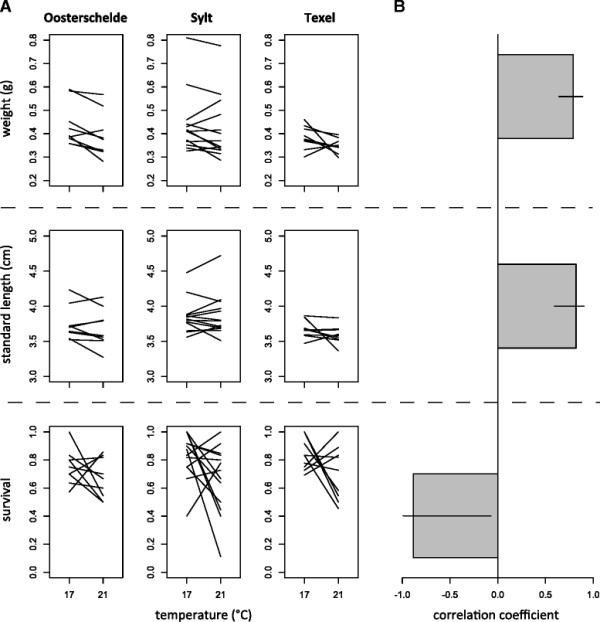
**Family-specific reaction norms and genetic correlations across environments. A** Family-specific reaction norms of weight, length and survival after infection with *Vibrio* sp. measured in three different stickleback populations. Lines represent different families connecting means in different temperature treatments. **B** Character state genetic correlations across thermal environments. All data were used for weight and length; only data from infected fish were used for survival.

We found similar slopes of reaction norms between families and no significant evidence for GxE interactions for weight and length (Figure 4A, Table 1). This finding was supported by high and positive genetic correlation coefficients, that despite being lower than one indicate weak GxE interactions in response to thermal stress (Figure 4B, Table 2). Pathogen resistance, on the other hand, showed larger variation between environments with many crossing reaction norms (Figure 4A). Survival also showed strong GxE interactions and significant negative genetic correlations (Figure 4B, Table 2). Although resistance at ambient temperatures may be only a weak predictor for resistance under thermal stress, increased resistance at elevated temperature of some families in all populations (Figure 4A) may suggest that population mean fitness may be partly resilient to environmental change. Also, the increase in within-population genetic variation at elevated temperature (Table 2) shows that there is evolutionary potential to facilitate a response to changing climate conditions.

**Figure 5:**
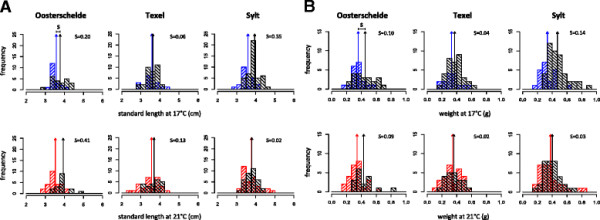
**Selection gradients of life history traits under pathogen induced selection.** Length **(A)** and weight **(B)** of dead fish (red bars = died at 17°C, blue bars = died at 21°C) and surviving fish (black bars) from different populations. Arrows show the means of the respective distributions marking the selection differential S (mean trait survived - mean trait dead) resulting from pathogen-induced mortality. Only data from infected fish were used for selective gradients.

Pathogen-induced mortality also exerted correlated selection on life history traits. At ambient temperature, surviving fish were significantly longer (parameter estimate length: 0.649, *P* <0.001, Figure 5A) and heavier (parameter estimate weight: 0.745, *P* <0.001, Figure 5B) than fish that died. At elevated temperature, differences between surviving and dead fish were smaller on average (parameter estimate length: 0.422, *P* = 0.007, parameter estimate weight: 0.382, *P* = 0.008), and selection differentials for body length increased in two out of three cases (Oosterschelde and Texel), whereas selection differentials for weight decreased uniformly in all populations.

## Discussion

In this study, we tried to connect the effects of elevated temperature on life history traits, pathogen resistance, and population-specific potential for evolutionary responses in marine sticklebacks. After only a short acclimation period at elevated temperature, we found impacts of thermal stress on fish weight and especially survival after infection. Furthermore, thermal stress tended to release cryptic within-population genetic variation (V_A_), which led to reduced genetic differentiation (Q_ST_) between populations. While life history traits showed positive genetic correlations between temperatures and only weak, non-significant GxE interactions, thermal stress led to negative genetic correlations across environments and strong GxE interactions in pathogen resistance. These trait-specific patterns indicate that evolutionary potential of life history traits (with positive genetic correlations across environments) can be reliably predicted from levels of standing genetic variation. For survival from infection, on the other hand, negative genetic correlations across environments indicate that current genetic variation in resistance may be a weak predictor for future resistance evolution, highlighting the crucial importance of disease in adaptation to new environmental conditions potentially favouring pathogens and parasites.

In general, thermal stress is likely to affect fish biochemistry, physiology and behaviour [[52]], and can negatively influence fish development and survival [[53]]. Interactions between elevated temperatures and decreased size and weight have been demonstrated in several studies [[32],[54],[55]], and it is believed that higher temperatures lead to lower food conversion efficiency in temperate sticklebacks, as energy is preferably used to maintain high metabolic rate [[55]]. Fish from Sylt were less affected by thermal stress throughout the experiment. This indicates the presence of different phenotypic optima as well as different thermal tolerances among the studied populations. Temperature preferences of sticklebacks for ideal growth were previously reported in the range between 15-18°C [[32],[33]], at 19°C [[56]], and also at 21,7°C [[57]], showing that thermal optima can differ substantially between populations. Temperate species are able to adapt and shift their thermal window through changes in mitochondrial densities as well as via other molecular and systemic adjustments [[58]]. The wide range of thermal optima reported in the literature in conjunction with our results on quantitative genetic differentiation (Figure 3) seem, however, to suggest that stickleback populations harbour substantial amounts of standing genetic variation and can adapt quickly to local environmental conditions.

The reduced sensitivity of the Sylt population can either be explained by increased tolerance due to phenotypic plasticity, or alternatively, by more favourable experimental conditions matching local conditions when compared to the other populations. We can assume that the allopatric populations from Oosterschelde and Texel were not only exposed to temperature stress but also had to cope with different water chemistry (our common garden experiment used seawater from Sylt). This might have led to less favourable overall conditions amplifying negative effects of temperature stress, thus resulting in a stronger decline of weight at elevated temperature. Although we showed that short-term acclimation to increased temperature negatively influenced fish development, previous studies have shown that long-term acclimation to higher temperatures (within the natural range) can potentially lead to enhanced development and increased growth rates [[58]-[60]]. The Sylt stickleback populations, on the other hand, continue to show decreased body size when exposed to higher temperatures throughout their lifetime [[35]], indicating that our thermal conditions exceeded their potential for acclimation, especially during early phases of development. Our results presented here thus support our previous results on a different trait. While we previously found differences in size during the whole ontogeny [[35],[61]], in the present study we did not observe significant influences of thermal stress on size in later life stages. Rather, we observed a response in weight. Although we could not track individual fish weight over time (i.e. we did not measure weight before the experimental treatment), we think it is highly unlikely that we accidentally produced our results by pre-existing bias. Since fish were randomly assigned to treatments, the chances of assigning heavier fish to the cold treatment in 23 out of 31 families used is very small (*P* = 0.003). Therefore, weight differences more likely represent effects of our treatment than pure chance. Additionally, we have previously shown that the efficiency of mitochondrial energy metabolism is reduced at 21°C [[61]], leading to higher energy demands and consequently lower resource availability. Increased use of reserves to cope with increased energy demand will lead to a drop in weight, and reduced growth can then be considered a secondary consequence of less available resources.

Our experiment also revealed that elevated temperatures have profound impacts on pathogen resistance of marine stickleback populations. Infected fish held in water at 21°C died faster and showed higher overall mortality than infected fish at 17°C. Suboptimal temperatures may influence innate immunity directly [[62]] or indirectly as a result of stress-linked overproduction of immunosuppressive cortisol [[63]]. It has also been shown that elevated temperature negatively affects the resistance of fish to diseases by affecting antibody production and leucocyte activity [[12],[64]]. Either way, stressed organisms are likely to be more susceptible to infectious diseases than non-stressed ones [[65]]. Increased susceptibility due to stress can additionally be amplified by increased virulence of pathogens at higher temperatures. Vibrios are generally favoured at warmer temperatures [[20],[21]], and the temperature optimum for *Vibrio tubiashii* is reported to be at 25°C [[66]]. We can thus assume that the elevated temperature of 21°C supported faster development and a higher concentration of bacteria in the fish resulting in higher pathogenicity. Additionally, surviving fish were significantly longer and heavier, with weight reacting faster to environmental change than length. Especially for weight, we consistently observed decreased selection differentials at elevated temperatures. The altered response to selection imposed by infection might indicate that responses of other fitness components also can change unpredictably in changing environments.

Such changes in responses to selection on single traits will ultimately also feed back on past and future evolutionary responses of the host. By using a Q_ST_-F_ST_ approach, we revealed that, especially for length and survival, the release of within-population genetic variance by environmental stress led to lower Q_ST_ values (even though between-population variances were not reduced). While the genetic components underlying phenotypic variation were less divergent for traits assayed at higher temperature (21°), thermal stress increased genetic variance (V_Animal)_ and heritability) for most of the selected traits (Table 2). The effects of environmental change on quantitative genetic differentiation have only rarely been addressed. Hoffmann and Merilä [[67]] summarized several outcomes of how unfavourable conditions can affect the genetic variation of a trait. First, stressful conditions can increase genetic variation in traits by increasing rates of recombination and mutation [[68]], by removing low fitness alleles due to selection [[69]], or because phenotypic differences among genotypes are only expressed as resources become limiting [[70]]. Second, genetic variance and heritability are decreased by increased environmental variation [[71]] or due to limited genetic potential of organisms under poor nutrition [[72]]. And third, stressful conditions have unpredictable effects on the genetic variation of a trait [[18],[73],[74]]. Our comparison of indices of population differentiation in quantitative traits (Q_ST_ of length, weight and survival) and neutral genetic divergence (F_ST_ of microsatellite markers) showed that divergent directional selection was the driving force for reaching different optima in length, weight and survival at 17°C in our stickleback populations (Q_ST_ > F_ST_).

Even if the comparison between Q_ST_ - and F_ST_ - values can reveal the presence and type of selection acting on traits [[75]-[78]], an essential requirement for evolutionary change is the amount of genetic variability expressed for the trait under selection [[79]]. We observed positive genetic correlations between character states of life history traits assayed at different temperatures. Although slopes of the genetic correlations across environments were significantly different than 1, family-specific reaction norms were rather flat, indicating that no significant genotype by environment interactions (GxE) affected life history traits. In contrast, pathogen resistance showed a negative genetic correlation across environments. This reversal of genetic effects is probably due to changing environment-dependent virulence mechanisms of the pathogen interacting differently with host genotypes, i.e. G_Pathogen_xG_Host_xE or (G_Pathogen_xE)x(G_Host_xE) interactions, rather than to pure host genetic effects, i.e. G_Host_xE interactions. On the other hand, some families within each of the populations showed improved performance at elevated temperature, which may lead to higher fitness in changing environments and adaptation of the population as a whole.

The maximal rate of adaptation is of crucial importance for estimating the ecological and evolutionary consequences of climatic warming. Populations need to shift the distribution of phenotypes to maintain optimal fitness in the changed environment [[14]]. Fast adaptation to rising temperature either requires short generation times or a substantial amount of standing genetic variation within populations [[80]]. Even though we found evolutionary potential in both environments, the negative genetic correlation in pathogen resistance between thermal environments demonstrates that adaptation to current conditions can, in certain cases, be a weak predictor for performance in changing environments. The negative genetic correlation across environments in survival might also have changed the selection gradients of associated life history traits, which might indicate that due to pathogen-induced selection, responses of other fitness components can be variable under unfavourable conditions.

## Conclusion

Our study shows that thermal stress can negatively impact life history traits and pathogen resistance of marine three-spined sticklebacks. Moreover, environmental stress led to negative genetic correlations across environments for pathogen resistance. Although we found evolutionary potential in both thermal environments, it is hard to predict the capacity for adaptation by selection of pathogen resistant genotypes from ambient conditions. While positive genetic correlations of life history traits between environments indicate similar responses to selection, the altered selective gradient imposed by infection shows that responses of vital fitness components can change unpredictably in changing environments.

## Availability of supporting Data

The data sets supporting the results of this article are available in the Pangaea repository http://doi.org/10.1594/PANGAEA.833937 [[81]].

## Competing interests

The authors declare that they have no competing interests.

## Authors' contributions

FSM wrote the manuscript, conducted the fish breeding as well as the experiment, dissected the fish, and was involved in designing the project, genotyping and performing statistical analyses. LNSS was involved in designing the project, running pilot studies and writing the manuscript. KMW was involved in designing the project, genotyping, performing statistical analyses and writing the manuscript. All authors read and approved the final manuscript.
